# Muscle strength and psychometric properties of the health-related quality of life in patients with soft tissue sarcoma resection in the thigh

**DOI:** 10.1016/j.clinsp.2023.100283

**Published:** 2023-09-30

**Authors:** Liza Furlan Ranzani Vitti, Carlos Eduardo Hideo Hanasilo, Cleide Moreira Silva, Mauricio Etchebehere

**Affiliations:** a Department of Orthopaedics and Traumatology, Faculdade de Medicina da Universidade de Campinas, Campinas, SP, Brazil; b Research Committee, Statistical Service, Faculdade de Medicina da Universidade de Campinas, Campinas, SP, Brazil

**Keywords:** Soft tissue sarcoma, Lower limb, Thigh, Rehabilitation, Muscle strength

## Abstract

**Objectives:**

The aim of this study was to evaluate the effect of STS resection in the thigh on MS and the HRQoL.

**Methods:**

Fourteen adults patients with STS in the thigh who underwent wide resection and limb preservation were evaluated. The patients were submitted to the Mini-Mental State Examination (MMSE). A hand-held dynamometer was used to measure the MS the flexors, adductors, abductors, and extensors muscles of the operated and non-operated thighs and between the dominant and non-dominant operated sides. The Musculoskeletal Tumor Society (MSTS) and Short Form Health Survey-36 (SF-36) questionnaires were applied to quantify the psychometric properties of the HRQoL. The data were submitted to statistical analysis using the Wilcoxon test (MS), and Mann-Whitney and Spearman correlation (MSTS and SF-36) (α = 0.05).

**Results:**

There was no significant difference in MS between the operated side and the non-operated side, and between the dominant and non-dominant operated side (*ρ* > 0.05). The MSTS presented a significant difference in the emotional acceptance for patients submitted to radiotherapy (*ρ* = 0.029). The SF-36 showed significant differences in the emotional aspect for patients submitted to chemotherapy (*ρ* = 0.027) and in the social aspect between the dominant and non-dominant operated side (*ρ* = 0.024).

**Conclusions:**

The HRQoL of adult patients is hampered after the treatment of STS even when MS is maintained.

## Highlights

• Supportive therapies are essential for maintenance of the muscle strength and quality of life.
• Some quality of life parameters are hampered after soft tissue sarcoma treatment.
• Soft tissue sarcoma treatment does not interfere with the functionality of the operated limb.

## Introduction

Sarcoma is classified into two types, bone sarcomas, and Soft Tissue Sarcomas (STS).^1^ The STS are ten times more frequent than bone sarcomas. The most common STS subtypes are undifferentiated pleomorphic sarcoma, liposarcoma, and leiomyosarcoma.^2^ These tumors arise from the mesoderm cells located in different body segments and often occur in the limbs of the adult population in the fifth decade of life or older.^1^,^3^ The thigh is the body segment more frequently affected in patients (44 %).^4^

Resection surgery with limb preservation is the choice in approximately 80 % of the cases in both malignant bone tumors and lower limb STS.^5^ The functional presentation of the patients after STS resection with limb salvage is much higher than patients who underwent amputation. The only exception is an amputation done below the knee.^6^ Treatment for patients with STS is focused on recovering the Muscle Strength (MS) and maintaining Health-Related Quality of Life (HRQoL). Furthermore, MS is an important component of health and HRQoL, since MS has an important role in the execution of many activities of daily living as well as is the most important predictor of function.^7^, ^8^, ^9^ In addition muscle weakness is related to disability^10^ and the decrease in MS can interfere in the emotional, social, and economic aspects.^11^

Aspects as function and psychometric properties, such as HRQoL, are evaluated using methods that quantify the patient discernment of the function of the operated limb and how it interferes with personal and professional life.^12^ The analysis of the functionality and HRQoL of adult patients who survived to a limb STS during childhood shows that they presented a high level of physical impairment and a low score on the HRQoL test.^13^ Furthermore, the impact of emotional and social behavior aspects of patients who underwent STS resection.^14^ However, according to our knowledge, there is no study that correlates the variation of HRQoL aspects with lower limb MS as well as in patients who survived to a limb STS during adult life (late diagnosis), not only in childhood.

Questionnaires are commonly used to assess the HRQoL of patients who have undergone surgical procedures for STS treatment, as they enhance the understanding of targeted therapy and/or the development of new target therapies in the management of STS.^15^,^16^ The literature emphasizes the MSTS^17^, ^18^, ^19^ and the SF-36^17^,^20^ as two reference questionnaires that effectively assess the HRQoL of these patients. Moreover, the MSTS can also evaluate lower limb functionality.^17^,^18^

The relationship of the HRQoL and functionality in patients who underwent STS resection with limb preservation is essential to evaluate how the intervention impacts the patient's daily life.^7^,^21^ Therefore, MS is an important parameter with regard to general health,^7^,^21^ since MS improves the functional capacity, which translates into improved HRQoL.^21^

Therefore, the aim of this study was to evaluate the impact of treatment of patients who underwent STS resection on the thigh on MS and HRQoL. The hypothesis would be that the patients who underwent STS resection on the thigh would have affected (i) the MS and (ii) the psychometric properties of HRQoL.

## Materials and methods

This cross-sectional and retrospective study involved a 2 × 2 factorial design for MS evaluation (dependent variable). The factors (independent variables) were the side of the thigh (two levels: operated and not operated), and the thigh function of the operated side (two levels: dominant and non-dominant) for each muscular group (flexors, adductors, abductors, and extensors). The Musculoskeletal Tumor Society Functional Scale (MSTS)^22^ and Short Form Health Survey-36 (SF-36)^23^ questionnaires (dependent variables) were applied to evaluate the psychological parameters of HRQoL according to factors (independent variables) adjuvant methods (two levels: radiotherapy and chemotherapy) and limb side operated (two levels: dominant and non-dominant).

This research has been approved by the IRB of the authors’ affiliated institutions (protocol: 64252516.3.0000.5404). The selected patients admitted to the Orthopaedic Oncology Service and underwent surgical treatment of STS between January 2002 and December 2017. The data from patients' medical records were collected and analyzed according to demographics, diagnosis and treatment (age, sex, histopathological diagnosis, muscle group and postoperative treatment).

The patients were selected according to the inclusion and exclusion criteria. The criteria of inclusion were patients with bone maturity who underwent STS resection in the thighs, a minimum of six months of postoperative follow-up, and a Mini-Mental State Examination (MMSE)^24^ score equal to or higher than 18. The exclusion criteria were late amputation of the resected limb, local recurrence of the tumor during the evaluation, disseminated diseases that avoid appropriate patient assessment, and patients currently undergoing chemotherapy or radiotherapy. The resection surgery (inclusion criteria) is the treatment of choice. This treatment represents approximately 80 % of the cases of malignant bone tumors and lower limb STS.^5^ The resection with limb preservation must obtain free margins and it can be associated or not to radiotherapy and chemotherapy.^5^

After patient eligibility, a cohort of 14 patients including six male and eight female were included in the study. All the patients signed an informed consent form to participate in the study. Then, the patients were submitted to MMSE to evaluate their cognitive capacity. No patient declined the participation and all patients finished the MMSE questionnaire.

### Muscle strength (MS) test

A hand-held dynamometer (Lafayette Manual Muscle Testing System, Lafayette Instrument Company, Lafayette IN, USA) was used to measure the isometric MS (kgf) of four muscular groups: flexors, adductors, abductors, and extensors, sequentially, of the hips of the operated and non-operated thighs. The patients were positioned in dorsal (flexors and adductors), ventral (extensors), and lateral (abductors) to evaluate the muscular groups. The hand-held dynamometer was adjusted perpendicularly to the area assessed and just above the patella. The anatomical axis of knee joint rotation (above the patella) was aligned with the axis of rotation of the dynamometer.

The patients were instructed to apply maximum strength in each requested movement for 10 seconds.^25^ The movement was repeated three times for each muscular group. The first movement was to familiarize the patient with the test. The last two movements were measured and documented for statistical analysis. The patient performed maximum voluntary isometric contractions. A difference of up to 10 % was accepted between the three measurements from the same muscular group. Differences higher than 10 % indicated the patient did not understand the requested movement. In this case, the patient repeated the movement after 60 seconds, and the value higher than 10 % was discarded from the calculation of the MS. The rest period between each repetition was 60 seconds to avoid muscular fatigue.

The measurement was performed by the same practitioner (L. F. R.) with expertise in muscle function of the thigh and to handle hand-held dynamometer. The test was conducted at approximately the same time of day (between 10 AM and 2 PM) to ensure MS test standardization.

### Psychometric properties

The MSTS adaptation and validation of the Portuguese version^22^ were applied to score for the lower limb. The MSTS questionnaire provides a functional assessment of six categories for patients following musculoskeletal tumor resection of limbs. The six categories are pain, function, emotional acceptance, support, walking and gait. The scale is 0‒5 with 0 being severe disabling pain and 5 being no pain. The total score of the MSTS was converted to a percentage (scale ranging from 0 to 100), with higher scores indicating better function.

The SF-36 questionnaire adaptation and validation of the Portuguese version^23^ was used to measure the HRQoL. It has 8 parameters such as physical functioning (10 items), mental health (5 items), general perception of health (5 items), energy and vitality (4 items), role limitations due to physical problems (4 items), role limitations due to emotional problems (3 items), social functioning (2 items), and pain (2 items). For each variable item scores are coded, summed, and transformed onto a scale from 0 (worst possible health state measured by the questionnaire) to 100 (best possible health state).

All the patients were asked to respond to the MSTS and SF-36 questionnaires on the same day before the MS test.

### Statistical analysis

Data collected from questionnaires (MSTS and SF-36) and MS tests were analyzed using non-parametric tests. The Mann-Whitney test evaluated the questionnaires. The Wilcoxon test was used to analyze the MS and compare both thighs. The Spearman correlation coefficient evaluated demographic characteristics and questionnaires (MSTS and SF-36). The significance level adopted was 5 % (α = 0.05). The statistical analysis was performed using the Statistical Analysis System software (SAS; Statistical Analysis System, 9.4, SAS Institute In., Cary, NC, EUA).

## Results

Demographic characteristics collected from patient's medical records and the percentage score of the MSTS of each patient are presented in Table 1. The mean age was 54.7 years (SD = 17.9), the Postoperative Time (POT) was 70.7 months (SD = 57.8), the mean MSTS score was 27.50 (SD = 2.3), and the mean MSTS percentage was 91.7 (SD = 7.6).

**Table 1:** Demographic characteristics, and MSTS score and percentage.

P	Sex	Age	Histopathological Diagnosis	Muscle Group	POT	Score	%
1	F	28	Low-grade fibromyxoid sarcoma	E	62	27	90
2	F	34	Alveolar sarcoma	A	139	29	83.3
3	F	40	High-grade pleomorphic sarcoma	E	43	23	100
4	F	44	High-grade pleomorphic sarcoma	A	10	27	90
5	F	49	Myxoid liposarcoma	E	55	30	96.7
6	F	54	Liposarcoma	A	179	28	80
7	F	63	Myxoid liposarcoma	A	183	29	100
8	F	85	High-grade pleomorphic sarcoma	E	12	30	80
9	M	32	Low-grade angiosarcoma	E	58	23	100
10	M	55	High-grade pleomorphic sarcoma	A	33	25	100
11	M	62	High-grade pleomorphic sarcoma	E	16	25	93.3
12	M	68	Myxofibrosarcoma	E	56	27	96.7
13	M	72	Myxofibrosarcoma	E	99	30	90
14	M	80	High-grade fusocellular sarcoma	E	45	30	83.3

P, Patient; F, Female; M, Male; POT, Postoperative Time; E, Extensor; A, Adductor.

The Wilcoxon test indicated no significant differences in MS between operated and non-operated sides for all muscular groups (*ρ >* 0.05) (Table 2). Also, the Mann-Whitney test presented no significant differences in the variation of the MS regarding the lower limb dominance (*ρ >* 0.05) (Table 2).

**Table 2:** Means (± SD) and ρ values of MS (kgf) of each muscular group between operated and non-operated sides (*n* = 14), and the variation of the MS between dominant (*n* = 9) and non-dominant (*n* = 5) operated sides.

	Flexor		Adductor		Abductor		Extensor	
Side	MS	ρ	MS	ρ	MS	ρ	MS	ρ
Operated	15.0 (18.7)	0.1873	9.8 (6.9)	0.9604	13.8 (9.2)	0.3757	12.0 (7.5)	0.1294
Not operated	14.3 (12.9)		9.3 (7.3)		15.8 (12.6)		9.4 (7.7)	
**Limb operated**								
Dominant	1.6 (9.3)	0.5050	0.6 (6.0)	0.5938	2.8 (7.9)	0.6892	0.3 (4.8)	0.1819
Non-dominant	1.0 (0.8)		0.4 (1.7)		0.7 (3.1)		6.6 (6.5)	

MS, Muscle Strength.

The Mann-Whitney test showed that the MSTS score and MSTS categories presented no significant differences between the patients who underwent and no- underwent each adjuvant method (radiotherapy and chemotherapy) (*ρ >* 0.05). The exception is the emotional acceptance parameter of patients who received radiotherapy (*ρ* = 0.029). Also, the dominance of the operated side did not influence the MSTS score (*ρ >* 0.05). Other parameters of MSTS (function, support, and walking capacity) were not significant since only one patient had a decreased score (Table 3).

**Table 3:** Means (±SD) and ρ values of MSTS according to adjuvant methods (radiotherapy and chemotherapy) and limb side (domimant and non-dominant) operated.

	n	Pain	ρ	Emotional acceptance	ρ	Gait		ρ	Score (%)
**RT**	Yes (6)	4.5 (0.8)	0.8209	3.0 (1.9)	0.0289	4.8 (0.4)	0.2446		89.4 (8.8)
	No (8)	4.2 (1.2)		4.9 (0.3)*		4.5 (0.5)			93.3 (6.7)
**QT**	Yes (3)	5.0 (0.0)	0.2027	4.3 (1.1)	0.9276	4.7 (0.6)	1.000		94.4 (9.6)
	No (11)	4.2 (1.1)		4.0 (1.7)		4.6 (0.5)			90.9 (7.3)
**DSO**	Yes (9)	4.8 (0.4)	0.0865	3.9 (1.7)	0.5326	4.7 (0.5)	0.8727		91.8 (8.0)
	No (5)	3.6 (1.3)		4.4 (1.3)		4.6 (0.5)			91.3 (7.7)

RT, Radiotherapy; QT, Chemotherapy; DSO, Dominant Side Operated. Asterisk indicate statistically significant difference between emotional acceptance within RT (*ρ* = 0.029).

Table 4 presents part of the SF-36 questionnaire data submitted to the Mann-Whitney test. There are no significant differences in most of the items in the patients who received or did not radiotherapy and chemotherapy, despite the operated limb (*ρ >* 0.05). However, the patients who did not undergo chemotherapy demonstrated the worst results in the social aspects limitation domain (*ρ* = 0.027). The same pattern of results occurred with the social aspects domain considering the limb dominance. The patients who had the dominant side preserved presented the lowest values (*ρ* = 0.024).

**Table 4:** Means (±SD) and ρ values of SF-36 according to adjuvant methods (radiotherapy and chemotherapy) and limb side (domimant and non-dominant) operated.

	RT			QT			DSO		
	Yes (*n* = 6)	No (*n* = 8)	ρ	Yes (*n* = 3)	No (*n* = 11)	ρ	Yes (*n* = 9)	No (*n* = 5)	ρ
**PF**	59.2 (38.6)	77.5 (21.2)	0.5582	86.7 (2.9)	65.0 (32.6)	0.8139	74.4 (28.0)	61.0 (35.1)	0.9464
**SAL**	50.0 (54.7)	62.5 (29.9)	0.7392	100.0 (0.0)*	45.4 (38.4)	0.0269	66.7 (41.5)	40.0 (34.9)	0.3025
**PAIN**	60.5 (38.0)	52.6 (32.8)	0.6488	76.7 (12.7)	50.4 (36.0)	0.2720	66.2 (21.5)	32.2 (41.4)	0.1069
**GH**	61.5 (19.5)	72.0 (19.2)	0.3646	73.3 (2.9)	65.9 (21.7)	0.8759	71.3 (20.7)	60.6 (16.3)	0.3491
**VT**	80.8 (12.8)	70.0 (21.5)	0.4708	83.3 (10.4)	72.3 (19.9)	0.3844	76.7 (16.8)	71.0 (23.0)	0.7349
**SA**	58.7 (28.4)	67.2 (27.5)	0.5949	83.3 (19.1)	58.2 (27.1)	0.1729	76.9 (21.1) ^#^	40.5 (21.6)	0.0235
**EAL**	66.7 (51.6)	75.0 (46.3)	0.8050	100.0 (0.0)	63.6 (50.4)	0.2750	88.9 (33.3)	40.0 (54.8)	0.0743
**MH**	85.7 (9.3)	79.5 (19.4)	0.6003	85.3 (8.3)	81.3 (17.4)	0.9270	82.3 (18.8)	81.8 (9.7)	0.4988

QT, Chemotherapy; RT, Radiotherapy; DSO, Dominant Side Operated; PF, Physical Function; SAL, Social Aspects Limitation; GH, General Health; VT, Vitality; SA, Social Aspects; EAL, Emotional Aspects Limitation; MH, Mental Health. Asterisk indicate statistically significant difference between social aspects limitation within QT (*ρ* = 0.0269). Number sign indicate statistically significant difference between social aspects within DSO (*ρ* = 0.0235).

The patient's age did not affect any parameter (MSTS and SF-36) (*ρ >* 0.05). The exception was the gait domain in MSTS (*ρ* = 0.0007). The Spearman correlation coefficient has shown that older patients presented significantly lower scores.

## Discussion

The first hypothesis of this study was rejected and the second hypothesis was accepted, since the STS resection on the thigh did not jeopardize the MS but affected the social aspects limitation and social aspects in patients who received chemotherapy and had dominant side operated, respectively.

Despite the several devices and methodologies used to measure the lower limb isometric MS, a hand-held dynamometry device was used because it has good reliability and validity in measuring of isometric the lower limbs MS.^25^ Furthermore, the methodology applied to measure of isometric MS was based on previous studies^25^ and the manufacturer instructions to promote a reliable result. The first noted aspect was the lack of difference in MS between the operated and the non-operated limb, even considering the side dominance. It was possible to observe that between the fourteen evaluated patients, nine presented the dominant side operated. Although the results do not show significant differences between dominant vs non-dominant sides operated groups for the MS (Table 2) and most domains (Tables 3 and 4), it is important to emphasize that humans have higher body consciousness and better mobility in the dominant side than a non-dominant side. Furthermore, the difference between the dominant and nondominant sides is clinically relevant since the healthy patients present an increase in MS on the dominant side (∼10 %)^26^,^27^ and, in most of cases, there is a decrease in MS after surgery.^26^

Several factors can explain the MS result, since many variables influence the MS, such as age, side, sex, and postoperative treatment.^23^ In the present study, patients operated on within a 16-year period (2002‒2017) were analyzed with at least 6 months postoperatively. Despite muscle recovery (motor and function) having been standardized between patients (data from questionnaires), the time of the muscle rehabilitation affects the MS, reducing the differences between operated and non-operated as well as dominant and non-dominant lower limb sides.^27^ The type of STS resection surgery must be considered in the MS analysis. A technical surgery with free margins allows functionality preservation of the lower limb muscles, preserving the MS. Moreover, this type of STS resection surgery can avoid local recurrence of the STS as well as some adverse effects, thereby favoring the prognosis.^9^ Another important point that can explain this result is that the patients compensate for the range of motion restrictions and MS, maintaining excellent lower limb function.^28^

HRQoL is essential to evaluate the results of tumor surgical treatments because, in general, the patients who underwent an STS resection exhibit a decrease in limb functionality, social function, and pain increase.^13^ In the present study, the MSTS showed no decrease in the values of the function, support, and walking capacity in patients submitted to adjuvant methods (radiotherapy and chemotherapy) and considering the limb side (dominant and non-dominant) operated. Only emotional acceptance was affected in patients who received radiotherapy. This adjuvant method had a negative effect on the emotional acceptance of the disease (Table 3). It is interesting that, in this study, the patients who received no radiotherapy were more satisfied with a high emotional acceptance score (4.9). This is primarily because of the high scores of pain relief, [4.2] gait maintenance, [4.5] and MSTS (93.3 %), which consequently improves emotional acceptance (Table 3). Radiotherapy can compromise tissue vascularization, resulting in susceptibility to skin complications, edema, and the formation of fibrosis.^16^,^29^ Although the patients in the present study did not show impaired gait (Table 3), these problems could result in local adherence and a decrease of the gait score, jeopardizing the HRQoL.^29^ Thus, special attention should be paid to the treatment of this possible bias, since radiotherapy is essential in STS treatment and it must not be interrupted to promote better local function.^29^

SF-36 is a comprehensive questionnaire with a broader spectrum of analysis,^10^ and it is a validated tool in medical research.^23^ Significant differences were observed in the social aspect's limitation according to chemotherapy and in the social aspects considering limb dominance (Table 4). These parameters refer to the physical and emotional health, connecting them to interferences in social activities (family and group inclusion) and routine work after the surgical procedure.^23^ Reulen et al.^30^ observed similar results in the adult population who had cancer during childhood. It is interesting to note that these results can be obtained also from patients who survived to a limb STS during adult life (late diagnosis), as shown by this study (Table 4).

The lowest score (45.4) for the social aspects limitation in the patients who had no received chemotherapy treatment (Table 4) in unexpected since the adverse effects of the chemotherapy are associated with decreasing the HRQoL.^30^ Several studies show that the number of cycles, hospitalization due to adverse effects, and long duration of post-chemotherapy side effects impacts negatively the HRQoL.^3^,^27^,^30^ In this study is hypothesized that, in the patients who received chemotherapy, the low number of these problems associated with the psychological therapy and education by the doctors and health-care workers about the management of adverse effects explain this result.

The patients who had the dominant side operated on presented the highest values (76.9) for social aspects of the SF-36 questionnaire (Table 4). This result is interesting because a drop in psychometric properties of the HRQoL is expected due to the fact that the dominant limb is in recovery treatment.^8^,^10^,^20^,^23^ However, this drop in HRQoL depends on some factors, such as the compromised limb and the employment performed by the patient. Limbs such as the hand and patients who perform physical activities dependent on the affected limb have compromised HRQoL.^8^ In the present study, the thigh did not directly affect the patients' daily tasks (data from patients' medical records). The body segment studied (thigh) associated with the maintenance of the MS (Table 2) can explain this result.

The results showed no correlation between HRQoL (MSTS and SF-36) and age (*ρ >* 0.05), corroborating with previous studies.^14^ The exception was the gait domain (MSTS), since that older patient presented the worst results (*ρ* = 0.0007). Some studies with general populations correlate the reduced HRQoL to the increase in age.^14^

This study showed the correlation of the functionality of patients (MS) with the selected criteria to the treatment and, consequently, HRQoL. It is important to emphasize that the correlation between MS and HRQoL demonstrated in the present study and in the literature does not represent causation. Further studies are needed to clarify this point. Furthermore, the results have significant clinical relevance, since they show that supportive therapies (psychological therapy e physiotherapy treatment; data from patients' medical records) are essential for the maintenance of the MS (Table 2) and to preserve the HRQoL, even when the patients are submitted to adjuvant methods (radiotherapy and/or chemotherapy) and have the dominant side of the thigh operated (Tables 3 and 4). The surgical intervention must preserve the function of the operated limb. The data must be analyzed considering the methodological limitations inherent to any cross-sectional and retrospective study. For instance, these studies could overlook some social and demographic data that could impact HRQoL, such as income, education, Body Mass Index (BMI), and so forth. The sample size must be also considered. Despite a higher frequency of STS than bone sarcomas, the STS is a low-prevalence disease and after patient eligibility, 14 patients were included in the study. Precision and accuracy of the data are affected by nonresponse bias, memory error, misunderstanding of questions, problematic definitions of terms, and processing errors, and not only by sample size. It is important to emphasize that the present analysis was rigorous to present direct results regarding MS and the HRQoL. To minimize bias (location, histological types, and so forth) that could interfere with the results, only patients with STS located in the thigh and a minimum of six months of postoperative follow-up were included in the present study.

## Ethical statement

The authors declare that the manuscript has been performed in accordance with the ethical standards in the 1964 Declaration of Helsinki. The patients and/or their families were informed that data from the research would be submitted for publication and gave their consent.

## Ethical review committee statement

The authors declare that the manuscript has been performed in accordance with the ethical standards in the 1964 Declaration of Helsinki.

## CONSORT statement rules

The authors declare that the manuscript has been performed in accordance with the CONSORT Statement rules.

## Author's contribution

Liza Furlan Ranzani Vitti: Conceptualization, Investigation, Methodology, Project administration, Writing original draft and Writing – review & editing.

Carlos Eduardo Hideo Hanasilo: Data curation and Formal analysis.

Cleide Moreira Silva: Data curation and Formal analysis.

Mauricio Etchebehere: Supervision and Validation.

## Declaration of Competing Interest

The authors declare no conflicts of interest.
